# Readiness to Provide Antenatal Corticosteroids for Threatened Preterm Birth in Public Health Facilities in Northern India

**DOI:** 10.9745/GHSP-D-20-00716

**Published:** 2021-09-30

**Authors:** Ankita Kankaria, Mona Duggal, Anshul Chauhan, Debarati Sarkar, Suresh Dalpath, Akash Kumar, Gursharan Singh Dhanjal, Vijay Kumar, Vanita Suri, Rajesh Kumar, Praveen Kumar, James A. Litch

**Affiliations:** a Post Graduate Institute of Medical Education and Research Chandigarh, India.; b All India Institute of Medical Sciences, Bathinda, Punjab, India.; c State Health System Resource Centre, Haryana, India.; d Survival for Women and Children (SWACH) Foundation, Panchkula, Haryana, India.; e Global Alliance to Prevent Prematurity and Stillbirth (GAPPS), Lynnwood, WA, USA.

## Abstract

**Introduction:**

In 2014, the Government of India (GOI) released operational guidelines on the use of antenatal corticosteroids (ACS) in preterm labor. However, without ensuring the quality of childbirth and newborn care at facilities, the use of ACS in low- and middle-income countries is potentially harmful. This study assessed the readiness to provide ACS at primary and secondary care public health facilities in northern India.

**Methods:**

A cross-sectional study was conducted in 37 public health facilities in 2 districts of Haryana, India. Facility processes and program implementation for ACS delivery were assessed using pretested study tools developed from the World Health Organization (WHO) quality of care standards and WHO guidelines for threatened preterm birth.

**Results:**

Key gaps in public health facilities’ process of care to provide ACS for threatened preterm birth were identified, particularly concerning evidence-based practices, competent workforce, and actionable health information system. Emphasis on accurate gestational age estimation, quality of childbirth care, and quality of preterm care were inadequate. Shortage of trained staff was widespread, and a disconnect was found between knowledge and attitudes regarding ACS use. ACS administration was provided only at district or subdistrict hospitals, and these facilities did not uniformly record ACS-specific indicators. All levels lacked a comprehensive protocol and job aids for identifying and managing threatened preterm birth.

**Conclusions:**

ACS operational guidelines were not widely disseminated or uniformly implemented. Facilities require strengthened supervision and standardization of threatened preterm birth care. Facilities need greater readiness to meet required conditions for ACS use. Increasing uptake of a single intervention without supporting it with adequate quality of maternal and newborn care will jeopardize improvement in preterm birth outcomes. We recommend updating and expanding the existing GOI ACS operational guidelines to include specific actions for the safe and effective use of ACS in line with recent scientific evidence.

## Abstract (precis)

In settings with limited resources that lack standards to ensure the quality of childbirth and newborn care, the use of antenatal corticosteroids is potentially harmful. Safe, effective use of antenatal corticosteroids requires providing standardized evidence-based practices and supportive supervision, training staff, and a facility-level actionable health information system.

## Teaser

**Key Findings:**

- Most of the 37 public health facilities in northern India that we assessed were not readied for providing quality care for threatened preterm birth and ensuring the safe use of antenatal corticosteroids (ACS).
- Deficiencies were identified in areas of evidence-based practices, competent workforce, actionable health information system, physical resources, and communication.
- The existing ACS guidelines were not up-to-date with current evidence and were not disseminated or implemented uniformly across facilities.
- Attention to accurate gestational age estimation and the quality of childbirth and preterm care were inadequate in all the facilities.

**Key Implications:**

- Increasing uptake of ACS for threatened preterm birth without providing adequate-quality maternal and newborn care and meeting essential preconditions outlined by World Health Organization recommendations will not improve preterm outcomes.
- Facility-level actions are needed for the safe and effective use of ACS in support of the delivery of quality care.

## INTRODUCTION

Globally, prematurity is a leading cause of death among children under the age of 5 years.^1^ In India, about 13% of babies are born preterm, which amounts to 3.5 million babies annually. Of these, 10% die due to complications of preterm birth and many of the survivors experience learning disabilities and hearing and vision problems.^2^^,^^3^

The use of antenatal corticosteroids (ACS) during preterm labor is acknowledged as one of the most effective interventions to improve preterm birth outcomes. ACS use has been widely studied in high-income countries where most facilities are equipped with adequate childbirth and preterm care and have the ability to estimate accurate gestational age. In a recent review of trials done mostly in high-resource settings, ACS use was associated with a reduction in respiratory distress syndrome (34%), intraventricular hemorrhage (45%), necrotizing enterocolitis (50%), and newborn mortality (31%).^4^ A lower rate of intensive care admissions (10%) and reduced cost of care (36%) are additional benefits of ACS.^3^^,^^5^ However, little evidence-based research is available on ACS use from low- and middle-income countries (LMICs) such as India.

In June 2014, the Government of India (GOI) published the *Use of Antenatal Corticosteroids in Preterm Labour (Under Specific Conditions by ANM): Operational Guidelines.*^6^ This was a pragmatic approach for India, where many women give birth at home or in facilities without advanced newborn care and without comprehensive emergency obstetric care. Although the primary focus of the operational guidelines was to empower auxiliary nurse midwives (ANMs) to provide ACS, the document was also intended for use by medical officers and staff nurses. In February 2015, the Antenatal Corticosteroid Trial,^7^ a multicenter trial of ACS use in 6 LMICs (including 2 sites in India) reported increases in newborn mortality and serious maternal infections with ACS use. Imprecise estimation of gestational age, inadequate newborn care, poor availability of postpartum maternal care, and poor assessment of imminent preterm birth have been proposed as factors that contribute to the increased mortality and morbidity with ACS use in LMICs.^6^^–^^9^ These findings raised international concern regarding the benefits versus risks of using ACS in peripheral settings in LMICs. Different authors have emphasized the need for adequately powered trials for establishing the safety and efficacy of ACS use in resource-limited settings.^3^^,^^8^^–^^10^

The World Health Organization (WHO) provided recommendations on threatened preterm birth in 2015, specifying essential preconditions for ensuring safe use of ACS.^11^ These preconditions include accurate assessment of gestational age, preterm birth imminent within 7 days, absence of evidence of maternal infection, and availability of adequate childbirth care and adequate preterm newborn care. Based on these guidelines, the WHO ACTION-I trial was undertaken to establish the safety and efficacy of ACS between 26 and 34 weeks of gestation in resource-limited settings, including India.^12^ In this multicountry trial, strict inclusion criteria were followed for selecting secondary/tertiary facilities and participants, and the findings of a low incidence of neonatal deaths and maternal infection provided reassurance about the benefits of dexamethasone in low-resource settings. Further, these findings re-emphasized that the benefits of ACS can only be achieved if WHO standard criteria are adopted for the appropriate selection of patients and if minimum standards of maternal and newborn care are provided.

ACS benefits can only be achieved with appropriate selection of patients and the provision of minimum standards of maternal and newborn care.

Health systems are also challenged by the resources required to adequately support the provision of ACS for threatened preterm birth. Two different multicountry analyses in 2015 and 2018 identified major bottlenecks in leadership and governance, health service delivery, health financing, health information system (HIS), essential medicine and technologies, community ownership, and partnership that hinder the uptake of ACS.^13^^,^^14^

This study was conducted in India to assess the current utilization and clinical practice of ACS use in threatened preterm birth at the health facility level. We assessed the training, knowledge, and attitudes of all levels of providers of obstetric care. The findings are intended to inform policy makers regarding facility readiness, recommend essential actions to support implementation, and catalyze the revision of the GOI operational guidelines to ensure safe and effective use of ACS.

## METHODS

To undertake this study, the role of key stakeholders including policy makers, professionals, and state representatives was critical. The Ministry of Health and Family Welfare, Child Health Division, GOI, established a project advisory group to approve the study framework and approach and review findings and current evidence to expand the scope of national operational guidelines for safe and effective use of ACS in threatened preterm birth. Representation included the Ministry of Health and Family Welfare, Global Alliance to Prevent Prematurity and Stillbirth, Haryana State Department of Health officials, Indian Council of Medical Research, National Neonatology Forum-India, Norway–India Partnership Initiative, Society for Women and Child Health, United Nations Children’s Fund (UNICEF), United States Agency for International Development, and leading Indian medical faculty.

### Study Design

A cross-sectional mixed-methods study was conducted in government health facilities in Hisar and Ambala districts of Haryana, India. The study assessed the facilities’ process of care and readiness for using ACS in threatened preterm birth. Health care providers (HCPs) were interviewed using a semistructured questionnaire to assess their knowledge and attitudes regarding ACS administration and evidence-based practices for ACS administration. Women were interviewed using a semistructured questionnaire to identify mothers’ perspectives on accessing care during preterm labor and their level of communication with HCPs.

### Study Sites

The selection of study districts was based on a purposeful selection process in consultation with the project advisory group to include 1 National Health Mission’s high-priority district (HPD) and 1 non-HPD. HPDs were identified based on process/outcome indicators for maternal health, child health, and family planning.^15^ Hisar as an HPD and Ambala as a non-HPD were selected with the concurrence of the Government of Haryana and respective district health authorities.

### Study Facilities and Population

Health facilities for assessment were selected in consultation with the local district health authorities based on the provision of delivery services and distance from district hospitals. All health facilities within the study districts had been classified according to the level of care. The primary health care centers (PHCs) and subcenters (SCs) were classified as primary care facilities, and community health centers (CHCs), subdistrict hospitals (SDHs), and district hospitals (DHs) were classified as secondary care facilities. A total of 37 health facilities, including 25 primary care (8 PHCs and 17 SCs) and 12 secondary care facilities (8 CHCs, 2 SDHs, and 2 DHs), were included in the study.

For the facility assessment component of the study, 17 SCs were excluded from 37 health facilities in the study districts because they were not providing delivery services.

For assessment of knowledge and attitudes regarding the safe use of ACS, a total of 88 HCPs involved in ACS administration, including medical doctors, staff nurses, and ANMs, were selected through convenience and purposive sampling.

To assess the care-seeking behavior of preterm delivered mothers who received ACS, 19 women up to 4 weeks postpartum and aged 15 years or older who delivered a preterm newborn were identified from clinical record registers.

HCPs involved in ACS administration were invited by trained field investigators to participate in the study through scheduled interviews. Eligible women were approached at the time of discharge. In addition, at the primary facilities, interviewers contacted health extension workers twice weekly to identify women who met the study criteria and had delivered at home. The health extension workers then informed these women either by telephone or in person about the interviewer’s visit. Consenting women were interviewed either immediately at a designated area of the health facility that was quiet and private, or at a more convenient time at the woman’s home. To avoid possible coercion, no financial incentives were provided. Inclusion criteria for facilities and participants are presented in Table 1.

**TABLE 1.** Study Tools, Inclusion Criteria, Facility/Participants, and Sample Size to Assess Use of ACS in Threatened Preterm Birth Public Facilities in Northern India

Study Tool	Inclusion Criteria	Facility Level/Participant	Sample Size
Facility assessment	District hospital, subdistrict hospital, community health center, primary health center, or subcenter in the study districts providing delivery services	District hospital	2
		Subdistrict hospital	2
		Community health center	8
		Primary health center	8
Health care provider interview	Health care providers involved in administration of ACS (medical doctor, staff nurse, or auxiliary nurse midwife)	Specialist doctor	6
		Medical officer	19
		Staff nurse	46
		Auxiliary nurse midwife	17
Clinical verification	Hospital records related to administration of ACS	District hospital	17
		Subdistrict hospital	5
Maternal care pathway	Women up to 4 weeks postpartum, aged 15 years or older, delivered preterm, and received at least 1 dose of ACS	District hospital	13
		Subdistrict hospital	6
HIS indicator extraction	Indicators related to ACS from facility reporting records	District hospital	2
		Subdistrict Hospital	2
		Community health center	8
		Primary health center	8

Abbreviations: ACS, antenatal corticosteroids; HIS, health information system.

### Study Framework

For a comprehensive assessment of ACS use, a framework for this study was developed from the *WHO Standards for Improving Quality of Maternal and Newborn Care in Health Facilities*.^16^ The study framework includes the 7 domains from the WHO standards for the facility process of care, and ACS use was assessed using 5 study instruments (Table 2).

**TABLE 2.** Study Instruments Mapped to Study Framework Domains to Assess Use of ACS in Threatened Preterm Birth in Public Facilities in Northern India

Framework Domain	Facility Assessment	Provider Interview	Clinical Record Verification	Maternal Recall Interview	HIS Indicator Report
Evidence-based practices	X	X	X		
Competent, motivated personnel	X	X			
Physical resources	X				
Actionable information system	X	X	X		X
Effective communication		X		X	
Respect and dignity		X		X	
Functioning referral system	X	X		X	

Abbreviations: ACS, antenatal corticosteroids; HIS, health information system.

### Study Instruments

Quantitative data were collected by using (1) a facility assessment tool; (2) a semistructured questionnaire for HCPs; (3) a clinical verification checklist and a case history extraction form; (4) a semistructured questionnaire for mothers; and (5) HIS indicator report summary. Data collection instruments were developed through a multistep process. Based on the study framework and the *WHO Recommendations on Interventions to Improve Preterm Birth Outcomes*,^11^ the scope of each tool was defined, and questions were drafted by the research team to include essential preconditions for facility readiness to provide ACS. These preconditions included accurate assessment of gestational age, preterm birth imminent within 7 days, absence of evidence of maternal infection, and availability of adequate childbirth care and adequate preterm newborn care. We also included all interventions from the 2015 WHO document that were related to the care of women with threatened preterm birth.^11^ The WHO-recommended interventions and resulting study framework used by the research team for assessment of clinical care elements are shown in the Box. The instruments were pilot-tested, and they were then further refined in terms of flow and content based on the feedback. The interviews were conducted orally by a trained research associate with a clinical background.

BOX **Framework for Assessment of Clinical Care Elements**

**Evidence-based care:** Practices assessed by focusing this domain to threatened preterm birth care in line with the *WHO Recommendations on Interventions to Improve Preterm Birth Outcomes.*^11^

- Availability of guidelines for ACS use: Availability in print/digital format of the national ACS guideline: *Use of Antenatal Corticosteroids in Preterm Labour Under Specified Condition by ANM: Operational Guidelines.*^6^
- Gestational age assessment: Different methods used by health care providers for estimation of gestational age such as use of fundal height, last menstrual period, and use of ultrasound. The most accurate estimate is from ultrasound scan before 24 weeks of gestation.^17^
- Identification of imminent preterm birth: Birth anticipated within 7 days (i.e., preterm labor, PPROM, severe preeclampsia/eclampsia, and antepartum hemorrhage).
- Adequate obstetric care: The basic emergency obstetric care services were assessed based on availability of drugs (parenteral antibiotics, magnesium sulfate, and oxytocin for management of infections, preeclampsia, and hemorrhage, respectively), manual removal of retained placenta, removal of retained products of conception, assisted vaginal delivery, and resuscitation with bag and mask of nonbreathing neonate. The comprehensive emergency obstetric care services were assessed based on obstetric surgery with anesthesia and blood transfusion availability.
- Adequate newborn care: Newborn care was assessed based on availability of newborn care corner, newborn stabilization unit, and special newborn care unit at the prescribed public health facilities and practices for thermal care; safe oxygen delivery; feeding support and infection management.
- Infection prevention and management in mother and newborns: This was assessed based on prescribed infection prevention interventions and clinical practices during antenatal, intranatal, and postnatal periods.^18^

**Competent and motivated workforce:** Availability of trained health care providers and their knowledge and attitude about providing adequate preterm care.

- Availability and training requirements: Availability of trained health care providers (specialists, medical officers, staff nurses, and auxiliary nurse midwives) as per Indian Public Health Standards.^19^^–^^23^
- Indications of ACS: ACS is indicated in preterm birth for gestational age 24 to 34 weeks expected within 7 days with one of the following: preterm labor, PPROM, severe pre-eclampsia/eclampsia, and antepartum hemorrhage.
- Preconditions: ACS use is recommended only when the following conditions can be met: threatened preterm birth between 24 and 34 weeks of gestation with accurate assessment of gestational age, preterm birth is considered imminent within 7 days, no clinical evidence of maternal infection, availability of adequate childbirth care (including the capacity to recognize and safely manage preterm labor and birth), and adequate preterm newborn care (including resuscitation, thermal care, feeding support, infection treatment, and safe oxygen use).
- Clinical parameters: Gestational age, fetal heart sounds, imminent preterm birth, cervical dilatation, PPROM, evidence of vaginal bleeding, and evidence of maternal infection.
- Signs of true labor: Regular uterine contractions, descent of presenting fetal part, and evidence of cervical shortening/dilatation.
- Minimum and maximum gestational age for ACS use: Minimum age is 24 weeks of gestation and maximum age is 34 weeks of gestation.
- Conditions that put woman at risk of preterm birth: Preterm labor, PPROM, severe pre-eclampsia/eclampsia, and antepartum hemorrhage.
- Route and dose of ACS administration: Administered intramuscularly and dose of dexamethasone is 4 doses of 6 mg at 12-hour intervals.
- Authority for ACS use by providers and existing supervision and monitoring practices.

**Actionable information system:** Availability of standard forms, review of completeness and accuracy of case sheets and registers of mothers who had received ACS and use of this data to improve care of mother and newborn. Documentation of ACS specific indicators as mentioned in Government of India 2014 ACS operational guidelines.

**Physical resources:** Availability of adequate physical environment, medicines, and equipment required for threatened preterm care.

**Effective communication:** Communication or counseling by health care providers and availability of IEC material on threatened preterm birth.

**Respect and preservation of dignity:** Communication or counseling by health care providers and availability of IEC material on threatened preterm birth.

**Functioning referral system:** Presence of an existing referral mechanisms for timely identification and safe referral with documentation of relevant information.

Abbreviations: ACS, antenatal corticosteroids; ANM, auxiliary nurse midwife; CPAP, continuous positive airway pressure; IEC, information, education, and communication; PPROM, preterm premature rupture of membranes.

### Data Entry and Analysis

The data were entered digitally using XLSForms and hosted on a server using KoBoToolbox.^24^ Quantitative data were analyzed in STATA 15.1,^25^ and descriptive statistics were applied.

### Ethics Approval and Consent of Participants

This project was reviewed and approved by the institutional review boards of the PGIMER, Chandigarh India (No. IEC-08/2016-491), and Project Concern International (No. 21). Letters of support were secured from all district offices and facilities where data were collected. We obtained written consent from potentially eligible and interested participants in their preferred language. We also informed them that their participation would be voluntary and there would be no professional or personal consequences or benefits of participation. Mothers were given the option to read or hear their consent form according to their literacy level. No financial incentives were provided.

## RESULTS

A total of 25 PHCs and 12 secondary health care facilities were included in the study. A total of 88 HCPs and 19 mothers were interviewed (Table 1). Additionally, 23 clinical verification cases and HIS indicators extraction assessments for the last 6 months were studied.

### Evidence-Based Practices

Table 3 describes the facility readiness practices and resources for ACS use in threatened preterm care as defined in the 2015 *WHO Recommendations on Interventions to Improve Preterm Birth Outcomes*.^11^ Emphasis on early ultrasound at <24 weeks of pregnancy to estimate gestational age in the form of an advisory or guideline was lacking at all levels. However, HCPs were using ultrasonography, last menstrual period, or fundal height for estimation of gestational age. Fundal height was being routinely used in 90% (18 out of 20) of the health facilities for gestational age estimation and 70% (14 out of 20 facilities) had a job aid for using fundal height for gestational age estimation. Among HCPs, 88% (n=78) reported using last menstrual period as a method for estimation of gestational age, 82% (n=72) reported using ultrasonography, and 65% (n=57) reported using fundal height.

**TABLE 3.** Facility Readiness Practices and Resources for ACS Use in Threatened Preterm Birth as Reported by Health Care Providers in Public Facilities in Northern India

	**Clinical Services**	**District Hospital, % (N=2)**	**Subdistrict** **Hospital, % (N=2)**	**Community** **Health** **Center,** **% (N=8)**	**Primary** **Health** **Center, % (N=8)**
Evidence-based practices					
Adequate gestational age assessment	Ultrasound fetal biometry	100	100	87.5	100
	Fundal height	100	100	87.5	87.5
	Advise for USG <24 weeks	100	100	100	100
Adequate obstetric care^a^	Parenteral antibiotics for maternal infection	100	100	100	100
	Parenteral magnesium sulfate for pre-eclampsia	100	100	100	100
	Parenteral oxytocic drugs for hemorrhage	100	100	100	100
	Manual removal of retained placenta	100	50	87.5	100
	Removal of retained products of conception	100	100	100	75
	Assisted vaginal delivery	50	100	50	50
	Resuscitation with bag and mask	100	100	100	100
	Obstetric surgery with anesthesia	100	100	NA	NA
	Blood transfusion facility	100	100	NA	NA
	Protocol for threatened preterm birth	0	0	0	0
Adequate preterm care	Measure labor room temperature	100	100	100	100
	Measure postnatal unit temperature	0	0	12.5	12.5
	Kangaroo mother care	100	50	37.5	25
Physical resources					
Essential medicine and functional equipment	Dexamethasone	100	100	100	100
	Oxytocin	100	100	100	100
	Magnesium sulfate	100	100	100	100
	Parenteral antibiotics	100	100	100	100
	Antihypertensives	100	100	100	87.5
	Antipyretics	100	100	100	87.5
	Oxygen	100	100	87.5	100
	Thermometer	100	100	75	100
	Ultrasound	100	50	0	0
	Fetoscope	50	50	100	75
	Dipstick for urine protein	100	50	100	75
	Phototherapy lights	100	100	75	62.5
	Pulse oximeters	100	100	75	50
	Radiant warmers	100	100	100	75
	Oxygen tubing	100	100	100	75
	Oxygen blender	50	0	0	0
	Nasogastric tube	100	100	100	87.5
	Container and cup	50	50	50	50
	Wall suction	50	50	25	62.5
	CPAP	50	0	0	0

Abbreviations: ACS, antenatal corticosteroids; CPAP, continuous positive airway pressure; NA, not applicable; USG, ultrasonography.

a Basic emergency obstetric care and comprehensive emergency obstetric care.

Basic emergency obstetric care services were reported to be available across all the facilities but a few SDHs, CHCs, and PHCs lacked services including manual removal of retained placenta, removal of retained products of conception, and assisted vaginal delivery. Comprehensive emergency obstetric care services were available at DHs and SDHs in both districts. However, none of the facilities had a comprehensive protocol for identification and management of conditions that put a woman at risk of threatened preterm birth such as severe preeclampsia and eclampsia, premature preterm rupture of membrane, antepartum hemorrhage, and spontaneous preterm labor. Most of the facilities were using partograph for monitoring of labor.

Newborn care corner, newborn stabilization unit, and special newborn care unit were present as prescribed by GOI guidelines.^26^ The availability of protocols for neonatal resuscitation, safe oxygen delivery, thermal regulation, feeding, and infection prevention were not uniform across facilities. The 2014 GOI operational guidelines had not been disseminated to the facilities.

### Competent and Motivated Workforce

The second subdomain assessed the knowledge and motivation of the workforce to provide adequate preterm care. All facilities reported a shortage of health care staff especially of physician specialists at CHCs, SDHs, and DHs and medical officers at DHs, SDHs, and PHCs. Only 23 out of 46 nurses (50.0%) and 8 out of 17 ANMs (47.1%) were trained in facility-based integrated management of neonatal and childhood illness. Among HCPs, ACS-related training was attended by 1 out of 6 specialists (16.6%), 3 out of 19 medical officers (15.7%), 3 out 46 staff nurses (6.5%), and 1 out of 17 ANMs (5.8%). The details of ACS-related knowledge about the clinical requirement for ACS use are presented in Table 4.

**TABLE 4.** ACS Guidelines Awareness, Training, and Knowledge Among Health Care Providers

	**Specialist,** **n (%)** **N=6**	**Medical Officer,** **n (%)** **N=19**	**Staff Nurse,** **n (%)** **N=46**	**ANM,** **n (%) ** **N=17**
Aware of 2014 Government of India ACS guidelines	4 (66.6)	10 (52.6)	3 (6.5)	6 (35.2)
ACS-related training attended	1 (16.6)	3 (15.7)	3 (6.5)	1 (5.8)
Minimum gestational age for ACS use	3 (50)	5 (26.3)	16 (34.7)	11 (64.7)
Maximum gestational age for ACS use	3 (50)	42.1 (8)	18 (39.1)	9 (52.9)
Correct route of administration	5 (83.3)	14 (73.6)	42 (91.3)	14 (82.3)
Correct dose of dexamethasone	2 (33.3)	8 (42.1)	25 (54.3)	41.1 (7)
Indications for ACS	1 (16.6)	0	2 (4.3)	1 (5.8)
Essential preconditions for ACS use	2 (33.3)	4 (21.1)	0	0
Ascertaining true labor	3 (50)	10 (52.6)	23 (41.3)	0
Conditions for risk of preterm birth	1 (16.6)	3 (15.7)	7 (15.2)	3 (17.6)
Critical parameters before ACS administration	0	1 (5.2)	0	0

Abbreviations: ACS, antenatal corticosteroids; ANM, auxiliary nurse midwife.

**FIGURE 1:**
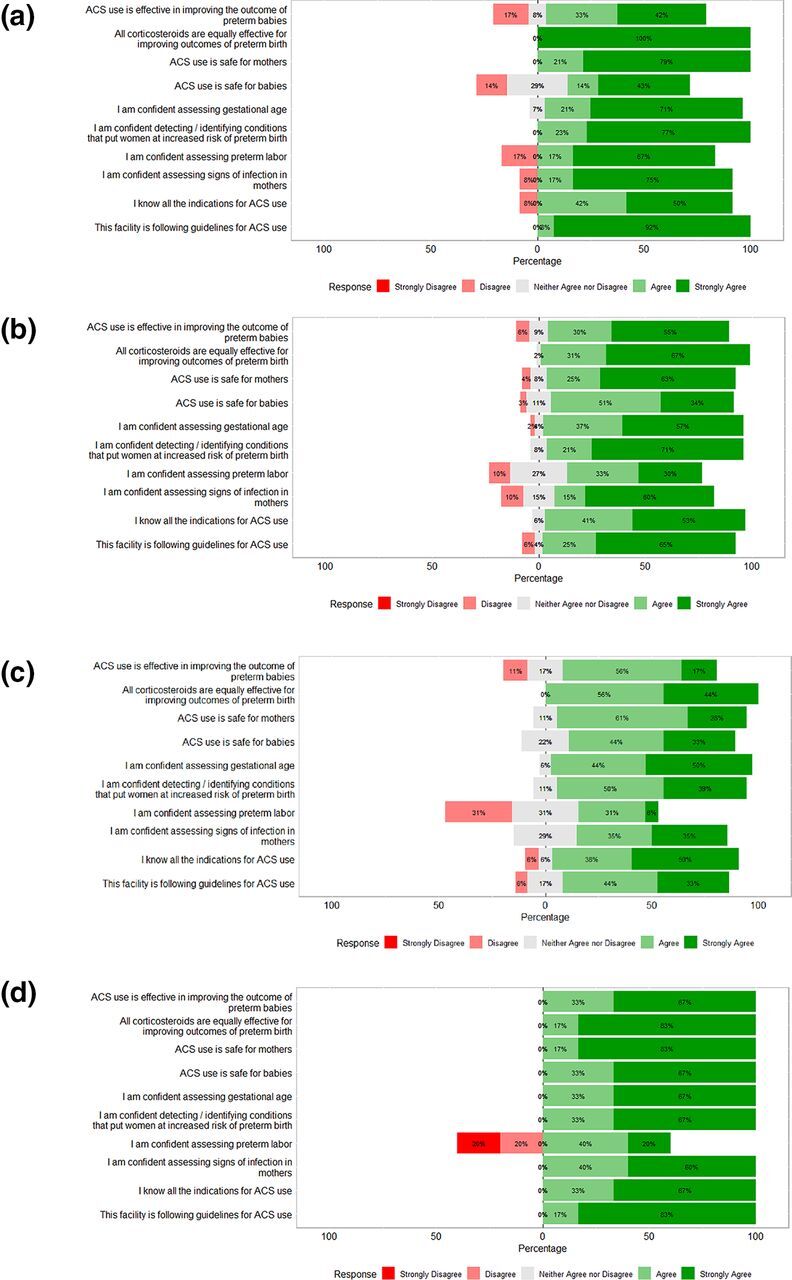
Attitudes of Health Care Providers Regarding Antenatal Corticosteroids Administration (a) Auxiliary Nurse Midwives; (b) Nurses; (c) Medical Officers; (d) Physician Specialists Abbreviations: ACS, antenatal corticosteroids.

The HCPs’ attitudes regarding the effectiveness of ACS and its safety and their confidence in ACS use were measured using Likert scales. A disconnect was observed between knowledge and attitude among all the HCPs; although HCPs reported they were confident about using ACS, their knowledge of essential clinical requirements was poor. This disconnect was observed more among ANM and staff nurses (Figure 1). In both study districts, a disconnect between attitude and knowledge was observed among all cadres. All cadres reported being confident regarding indications for ACS, identification of signs of preterm labor, and identification of conditions that put women at risk of preterm birth, but they scored poorly on knowledge assessment on these parameters/domains.

A disconnect was observed between knowledge and attitude; although HCPs reported confidence about using ACS, their knowledge of essential clinical requirements was poor.

### Decision for ACS Administration

A total of 76 of 88 HCPs (86.4%) reported that decisions for ACS administration were made mainly by doctors; 29 of 88 HCPs (32.9%) reported that the decision was made by staff nurses; and 7 of 88 HCPs (7.9%) reported that the decision was made by ANMs.

### Supervision or Monitoring of HCPs

None of the facilities had a routine audit or supportive supervision strategy in place to ensure safe and effective use of ACS.

### Physical Resources

Dexamethasone, oxytocin, magnesium sulfate, and parenteral antibiotics were available in most of the facilities, except dexamethasone in 1 CHC and 1 PHC in the past 6 months. However, antihypertensives, oxygen, and antipyretics were not available at some of the facilities. Ultrasound machines were available only at DHs and 50% of the SDHs. The necessary equipment for thermal regulation, oxygen delivery, and feeding were either unavailable or nonfunctional at the time of facility assessment. The remaining equipment, such as blood pressure machine, stethoscope, and baby weight scale, were available in all health facilities. All facilities had a continuous water supply and power backup.

### Actionable Health Information System

The clinical record documentation of 19 mothers who had received ACS in health facilities was assessed. Of these mothers, 79% were given ACS between 24 and 34 weeks, and 21.1% were given ACS after 34 weeks. In all the cases, ACS-specific indicators as per the 2014 guideline were not recorded or monitored. Moreover, indicators on the quality of ACS use, such as administration beyond 34 weeks and use of ultrasound for gestational age estimation, were lacking in the existing guidelines. Moreover, these facilities lacked monitoring of ACS-specific facility readiness in terms of existing infrastructure, human resources, clinical practices, and documentation of relevant information.

### Communication, Respect, and Dignity

We interviewed 19 mothers who had a preterm delivery in the previous 1 month and those who had received ACS in an SDH or DH regarding their experiences with the care provided. More than two-thirds (68.4%) of the mothers were informed about recognizing true labor, and 4 out of 19 mothers (21.2%) were informed about the risk of preterm labor. The newborn’s condition was explained to 58% of the mothers, and 16% were informed regarding ACS before administration. Although danger signs of pregnancy were printed on the antenatal maternal child protection card, ACS-specific information in the vernacular language was not available.

### Functioning Referral System

Out of 19 women, 11 were referred for care. Eleven women were referred by an HCP and 8 others reached secondary care facilities on their own. The reasons for referral were pain, bleeding, leakage, and weakness. Referrals were made mainly to DHs that the patient could reach within 30 to 60 minutes using the state ambulance services. Eleven mothers were provided an ambulance for referral. Referral slips were given to 9 women. A separate referral card was made for newborns in all DHs, 1 out of 2 SDHs (50%), 6 out of 8 CHCs (75%), and 3 out 8 PHCs (37.5%). The documentation of information about ACS use on referral slips/cards was poor.

## DISCUSSION

This study is the first systematic analysis of facility process of care in the context of the 2014 GOI guidelines to provide ACS for threatened preterm birth in public health facilities. Our findings suggest that most of the facilities were not equipped for providing quality care for threatened preterm birth and ensuring safe use of ACS. The facilities that were deficient in the quality of care domains of evidence-based practices, competent workforce, actionable information, physical resources, and communication and respect failed to fulfill the preconditions outlined in the 2015 WHO recommendations.^11^ Overall, the study facilities lacked monitoring of ACS-specific facility readiness.

Our findings suggest that most of the facilities were not equipped for providing quality care for threatened preterm birth and ensuring safe use of ACS.

In comparisons across levels of facilities, DHs were better equipped with infrastructure and trained human resources. The reported use of different methods (ultrasonography, last menstrual period, and fundal height) by HCPs for estimating gestational age indicates the need to emphasize the importance of standardizing the accurate estimation of gestational age by ultrasonography within 24 weeks of pregnancy. Although most of the facilities were using a partograph for monitoring labor, the completion of the form was not assessed. The basic emergency obstetric care packages in SDHs, CHCs, and PHCs need to be strengthened in the guidelines. A comprehensive protocol for the identification and management of threatened preterm birth should be developed, tested, and implemented in the health facilities.^11^^,^^13^^,^^14^

Similarly, ACS clinical knowledge was better among doctors at the secondary care level and poorest among ANMs. But there is a shortage of doctors, especially physicians and specialists at CHCs, SDHs, and DHs and medical officers at DHs, SDHs, and PHCs. Therefore, a cadre-specific training curriculum updated with the latest evidence on identification and management of threatened preterm birth should be in place to train HCPs based on their assigned level of facility. Additionally, to address the shortage of health care staff, regular recruitment should be in place to ensure continuous service delivery. Periodic audits and supportive supervision should also be in place to sustain the quality of care. Decision for ACS administration should only be made either by a medical officer or a staff trained in the management of threatened preterm birth. Health systems can be further strengthened to provide timely ACS at secondary-level facilities through strict monitoring of the supply of ACS and by adequate inventory management. The necessary equipment for thermal regulation, oxygen delivery, and feeding should be made available or made functional.^27^^–^^29^ A regular audit should be in place for the availability of equipment and medicines essential for providing threatened preterm birth care.

Timely referral with details of the diagnosis, investigations, treatment given (i.e., ACS time and dose), and reason for referral of the mother as well as the newborn can facilitate appropriate and prompt decision making and improve maternal and newborn outcomes.^30^ Communication and counseling at the time of referral and treatment constitute an important aspect of health care delivery, build confidence in the health system, and improve patient satisfaction. This need was reflected in the present study because mothers were not informed about labor signs, danger signs, the need for ACS administration, and newborn condition. The availability of information, education, and communication material in vernacular language will help patients and caregivers to be aware of and communicate danger signs or conditions leading to preterm labor and indications for use of ACS.

This assessment of facility readiness to provide ACS for threatened preterm birth was aligned with the WHO quality of care framework, in contrast to previously published assessments.^13^^,^^14^^,^^31^ In 2014, the WHO completed a multicountry assessment limited to the coverage of ACS.^28^ Other studies have looked at systematic targeted approaches to the strengthening of health systems, with a focus on overcoming specific bottlenecks for the highest impact interventions.^13^^,^^32^ In one multicountry assessment, policy makers were interviewed to assess compliance with WHO recommendations for use of ACS.^14^ In a multicountry analysis of 11 countries (including 3 sites in India) in 2015, 9 countries documented major bottlenecks in health system building blocks under ACS-specific health service delivery, HIS, and essential medicines.^13^ These mainly included lack of clear guidelines and training; limited capacity in gestational age estimation and identification of threatened preterm birth; shortage of HCPs at high cadre and discrepancies between cadres who were prescribing authority and cadres who were care providers; deficiency in data on ACS coverage, use, and outcome; lack of critical reviews of ACS use in clinical audits; lack of national essential medicines listing; delays due to referral; and lack of supervision, mentoring, and quality improvement systems.^13^ A policy implementation analysis of ACS use in 7 sub-Saharan African countries highlighted a lack of emphasis on essential preconditions for ACS use such as accurate estimation of gestational age, critical window period of 24–34 weeks for ACS administration, identification of threatened preterm birth, and contraindications for ACS use.^14^ Another study on quality of maternal and newborn care in public health facilities (DHs, PHCs) in Bihar reported gaps in structural capacities such as availability of basic infrastructure, essential equipment and supplies, and adequate staff.^33^ Similar to the present study, findings from Latin America on knowledge of HCPs regarding ACS use reported the need to improve knowledge on the indication, benefits, and dose regimen of ACS.^34^ A recent review from LMICs concluded that ACS risks and benefits may change if the health system is too weak to support preterm deliveries and subsequent preterm care.^35^ The principle of “Do No Harm” has been invoked by authors assessing ACS use in LMICs.^10^^,^^14^ An editorial emphasized an urgent need for advocacy for the safe use of ACS by maternal and newborn health experts.^10^

In 2020, WHO published a multicountry double-blind randomized trial in Bangladesh, India, Kenya, Nigeria, and Pakistan to assess the safety and efficacy of dexamethasone in women in hospitals in low-resource countries who were at risk for early preterm birth.^12^ The study provided robust evidence that ACS for threatened preterm birth in facilities that met the 2015 WHO recommendations for ACS use only under certain conditions, including the accurate assessment of gestational age, imminent preterm birth, the absence of maternal infection, and adequate care for childbirth and preterm newborns resulted in significantly lower risks of neonatal death alone and stillbirth or neonatal death without an increase in the incidence of possible maternal bacterial infection. For ensuring the safe and effective use of ACS in threatened preterm birth, facility readiness to meet the preconditions outlined by the 2015 WHO guidelines is an essential prerequisite.^11^ The existing 2014 GOI guideline should be updated with recent evidence and expanded to emphasize accurate gestational age estimation, ACS-specific readiness for infrastructure, human resources, clinical practices, and monitoring indicators. Assessments based on a quality of care framework for essential processes for providing care in facilities may prove beneficial in quality improvement activities targeting ACS use.^36^ A quality improvement initiative for ACS use comprising a technical update followed by an audit of and feedback on ACS data led to an increase in ACS coverage, knowledge score, and confidence of HCPs, as well as a complete recording of ACS data in the Philippines and Cambodia.^37^ Our study identified the need for establishing a quality improvement and monitoring system for ensuring appropriate use of ACS in line with the 2015 WHO recommendations.

For the safe and effective use of ACS in threatened preterm birth, facility readiness to meet the preconditions outlined by the 2015 WHO guidelines is an essential prerequisite.

**FIGURE 2:**
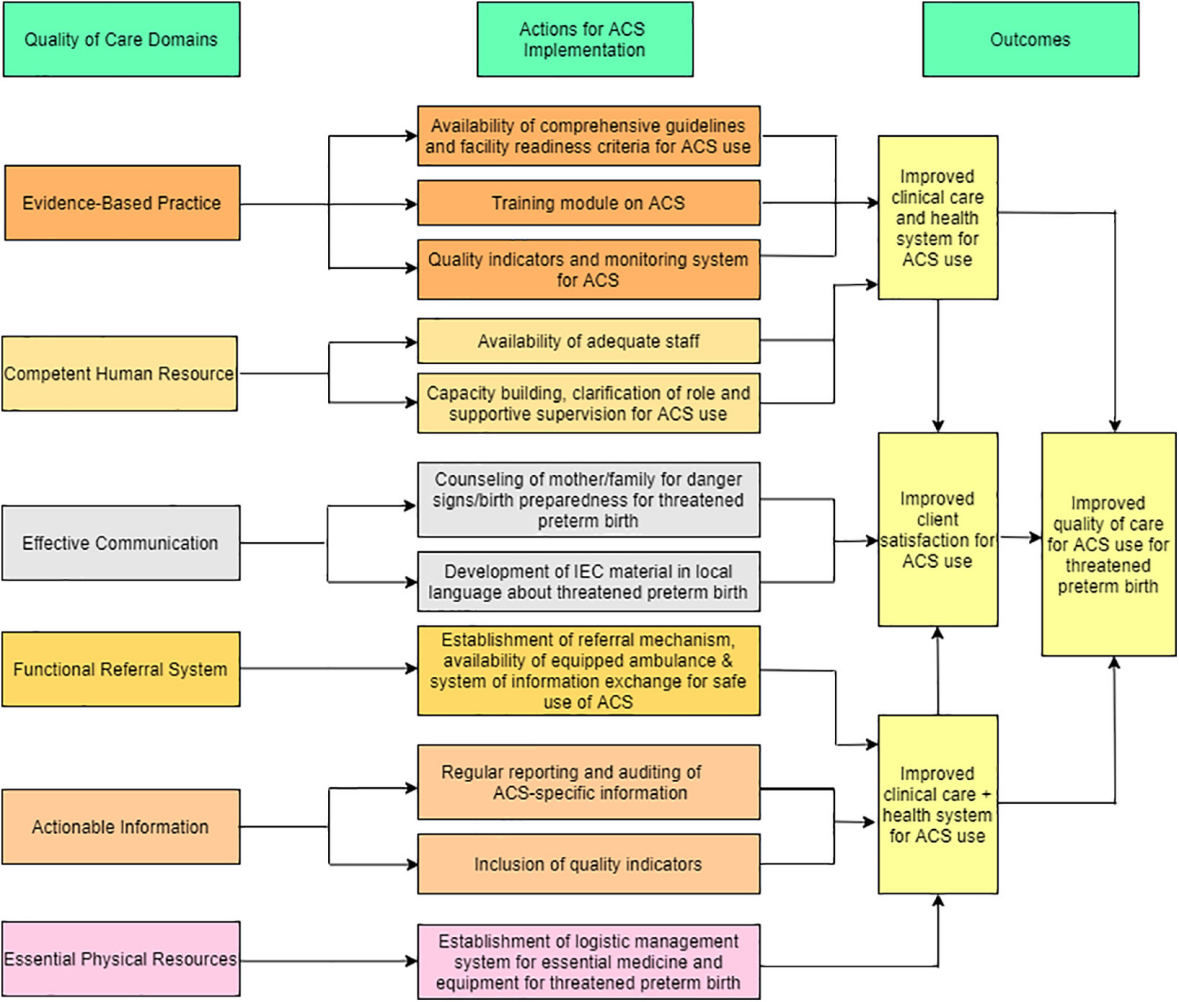
Proposed Actions and Outcomes for the Safe and Effective Use of Antenatal Corticosteroids for Threatened Preterm Birth Abbreviations: ACS, antenatal corticosteroids; IEC, information, education, and communication.

Suggested actions for ensuring safe and effective use of ACS include (1) expanding the scope of existing guideline from preterm labor to threatened preterm birth; (2) developing facility readiness criteria; (3) regular training and audit of HCPs; (4) expanding existing indicators on ACS coverage to indicators on the quality of ACS use; (5) developing efficient logistic management; (6) strengthening of existing referral system; (7) improving counseling practices of HCPs; and (8) developing information, education, and communication material on preterm birth care in the local language and engaging mothers/family in childbirth care/process (Figure 2).

### Limitations

For this assessment of the readiness of facilities to provide ACS, we followed the WHO quality of care framework^16^ with an emphasis on clinical requirements^11^ from a provider/facility viewpoint with observational validation when possible. Additional elements of the analysis focusing on the experience of care included the perspectives of preterm delivered mothers were more cursory. For this report, the focus was specifically on providers of ACS services and their clients (preterm-delivered women) and not on facility-in-charges or other facility staff.

We did not include the tertiary care level because the national guidelines are generally implemented by individual states up to the district level. The 2014 GOI guideline did not stratify the strategies for ACS use at respective primary, secondary, and tertiary care levels and did not include the facility readiness for safe and effective use of ACS.

Tertiary care centers that are referral centers do not follow specific guidelines, and treatment is physician specific based on the condition of the patients. Most of these centers are either medical colleges or advanced institutes that have trained staff, and they are well equipped in providing very advanced maternal and newborn services.

Another limitation of this study is the generalizability of the finding to other states in India. Moreover, private health facilities were not included in the study. However, this study can be replicated in other states and the findings can be used for the expansion of existing guidelines. The data presented on evidence-based practices were self-reported by HCPs and could not be verified by observation.

## CONCLUSIONS

Health facilities at primary and secondary levels lack facility readiness to provide quality of care for threatened preterm birth and safe use of ACS for threatened preterm birth. This study suggests a need to strengthen the existing health system by improving advocacy for ACS programs and quality of health care delivery, training of HCPs, and developing an actionable information system. Merely increasing uptake of any single intervention^38^ such as ACS without supporting it with adequate quality of maternal and newborn care and meeting essential preconditions in line with the 2015 WHO recommendations^11^ will not result in improving preterm outcome. Such improvement requires functioning health facilities and integrated planning and delivery of efficient, effective, and quality care to mothers and children^39^ based on up-to-date national policy and guidelines for ACS use that are evidence-based and directed at all levels of facilities and cadres of HCPs.

## Notes

**Peer Reviewed**

**First published online:** July 26, 2021.
